# Hepatitis B virus functional cure in persons with HIV: what are the predictors and which novel markers are useful?

**DOI:** 10.1097/COH.0000000000001002

**Published:** 2025-12-19

**Authors:** Lorin Begré, Fabien Zoulim, Anders Boyd

**Affiliations:** a Department of Infectious Diseases, Inselspital, Bern University Hospital, University of Bern, Bern, Switzerland; b Peter Medawar Building for Pathogen Research, Nuffield Department of Medicine, University of Oxford, Oxford, UK; c UMR PaThLiv U1350, Inserm, Université Claude Bernard Lyon 1; d Lyon Hepatology Institute, IHU EVEREST; e Department of Hepatology, Hospices Civils de Lyon, Lyon, France; f Amsterdam Institute for Immunology & Infectious Diseases, Infectious Diseases Program; g Amsterdam UMC, Location University of Amsterdam, Department of Infectious Diseases, Amsterdam, the Netherlands

**Keywords:** biomarkers, co-infections, HBsAg loss, hepatitis B virus, HIV

## Abstract

**Purpose of review:**

For individuals with hepatitis B virus (HBV), hepatitis B surface antigen (HBsAg) loss is associated with substantially decreased risk of liver-related morbidity and mortality. In recent years, many determinants of HBsAg loss have been investigated in several studies involving persons with chronic HBV infection living with and without HIV. The purpose of this review is to summarize factors that could help predict HBsAg loss in persons with HIV (PWH).

**Recent findings:**

Rates of HBsAg loss can be higher in PWH with HBV compared to those without HIV, which has been partially attributed to immune reconstitution after starting antiretroviral therapy. In recent years, quantitative HBsAg (qHBsAg) levels were identified as the most important single serum marker predicting HBsAg loss. Other viral markers, such as hepatitis B core-related antigen, circulating HBV RNA, and immunological markers (i.e., quantitative hepatitis B core antibody, assessment of HBV-specific immune responses, peripheral blood mononuclear cells phenotypes), might also help predict HBsAg loss in PWH with HBV, particularly for certain sub-populations.

**Summary:**

Low qHBsAg before or fast qHBsAg declines after initiating potent anti-HBV therapy has been identified as the most reliable predicting serum marker. Other markers might be useful in certain sub-populations and clinical situations.

## INTRODUCTION

With approximately 8% of all persons with HIV (PWH) also living with chronic hepatitis B, hepatitis B virus (HBV) co-infections are a major cause of morbidity and mortality in the era of widely available effective antiretroviral therapy (ART) [1]. ART containing either tenofovir disoproxil fumarate (TDF) or tenofovir alafenamide (TAF) suppresses HBV viral replication in the majority of persons, but requires a high level of adherence to treatment [2]. Despite this level of suppression, the risk of progression of liver disease and development of hepatocellular carcinoma (HCC) remains elevated [3]. In PWH, regression of liver fibrosis during antiviral therapy appears to be less pronounced compared to persons without HIV (PWoH) [4,5].

To eradicate HBV infection from the individual, production of covalently closed circular DNA (cccDNA) in the nucleus of the hepatocytes needs to be halted [6]. Currently, there are no antiviral agents targeted toward cccDNA production, which makes this an unrealistic therapeutic goal. The sustained loss of hepatitis B surface antigen (HBsAg) is associated with improved clinical outcomes and can occur in persons with HBV [7,8^▪▪^]. It is usually durable as HBsAg seroreversions are a relatively rare event [9]. As such, HBsAg loss is considered a more relevant and realistic therapeutic goal. However, viral DNA also integrates into the host genome during the HBV replication process. In addition to cccDNA, integrated DNA (iDNA) serves as template for HBsAg expression and may consequently impact HBsAg loss rates [10^▪^].

Although antiviral therapy allows potent suppression of circulating virus, HBsAg loss occurs infrequently with less than one event per 100 person-years in PWoH [11^▪^]. Interestingly, substantially higher rates of HBsAg loss have been described among PWH [12^▪^,13^▪^,^14^,^15^,16^▪^,^17^,18^▪^]. In recent years, multiple studies have investigated the determinants of HBsAg loss in persons with and without HIV. In the present review, we aim to provide a comprehensive overview on the most recent data on predictors of HBsAg loss in PWH and HBV, with particular focus on more novel serum biomarkers. 

**Box 1:**
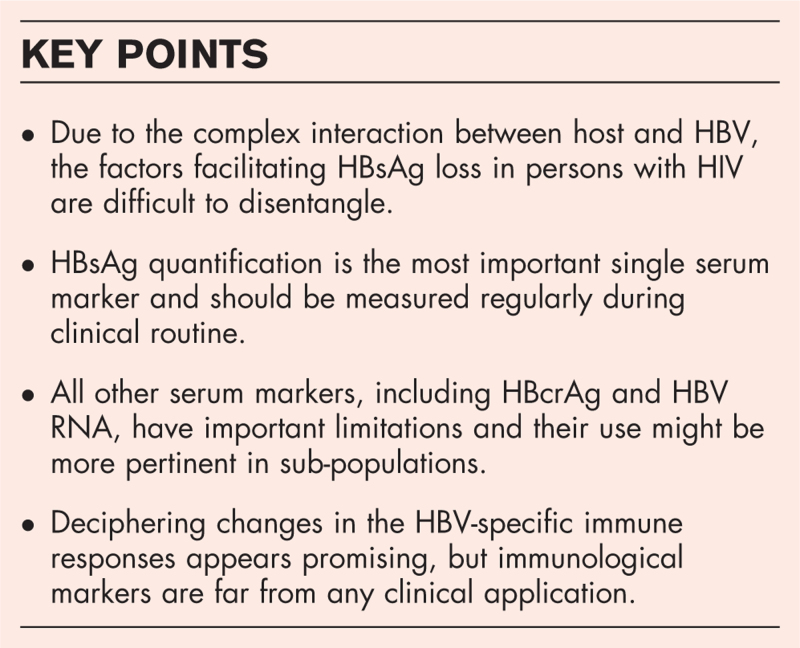
no caption available

## HOST AND VIRUS DETERMINANTS OF HBsAg LOSS IN PERSONS WITH HIV

Multiple factors may contribute to the higher probability of HBsAg loss in PWH compared to PWoH (Table 1). This highlights the complex interaction between antiviral host immune responses and the viral life cycle of HBV.

**Table 1:** Predictors of functional hepatitis B virus cure in persons with HIV

Predicting factors		References reporting an association with HBsAg loss
Host factors	Low CD4^+^ cell counts at start of ART	[19,20^▪^,^21^]
	No occurrence of AIDS-defining conditions	[14]
	Extremes of age	Younger age [24^▪▪^,^27^,^31^], older age [25,26], both extremes of age [28^▪^]
	Female sex	[18^▪^,^19^,^27^]
Antiviral therapy	Tenofovir alafenamide-containing ART	[24^▪▪^]
Virologic determinants	Liver inflammatory activity	Higher baseline ALT levels [18^▪^,^24^^▪▪^], lower liver stiffness [24^▪▪^], hepatic flares after start or interruption of ART [22^▪▪^,^23^,^24^^▪▪^]
	HBV genotypes A and B	[33,34^▪^]
	Lack of mutations in the *precore* gene	[37,38]
	Lack of viral integration	[10^▪^,54^▪^]
Novel serum markers	Quantitative HBsAg levels	Low baseline levels [12,18,20^▪^,^24^^▪▪^,^26^,^42^–^49^], declining levels [26,45,50^▪^,51^▪^,^52^,53^▪^,64^▪^]
	HBcrAg levels	Low baseline levels [18^▪^,^69^,^80^], declining levels [64^▪^,68^▪^,^78^]
	Circulating HBV RNA levels	Low baseline levels [18^▪^,^80^],declining levels [64^▪^,^78^]
Immunological markers	Quantitative HBcAb levels	low baseline levels [92,93^▪^], declining levels [90,91]
	IP-10 levels	High baseline levels [95,96], increasing levels [97^▪^]
	Lower baseline soluble PD-1 levels	[21]
	HBV-specific T-cell responses	High T-cell activation and proliferation [98^▪▪^], different T-cell immune profiles [99,100,101^▪^]
	HBsAb-specific B-cells	[102^▪^]

ART, antiretroviral therapy; HBcAb, hepatitis B core antibodies; HBcrAg, hepatitis B core-related antigen; HBsAb, hepatitis B surface antibodies; HBsAg, hepatitis B surface antigen; HBV, hepatitis B virus, IP-10, interferon-gamma inducible protein 10; PD-1, programmed death-1.

As the highest rates of HBsAg loss have been found among PWH initiating ART at low CD4^+^ cell counts or more advanced HIV infection [16^▪^,^19^,20^▪^,^21^], immune reconstitution seems to be an important driver of HBsAg loss. Nevertheless, more recent studies involving PWH/HBV from Europe did not confirm the association between lower CD4^+^ cell counts or the occurrence of AIDS-defining conditions and higher probability of HBsAg loss [12^▪^,^14^,18^▪^]. A recent study including PWH/HBV from China have described the association between hepatic flares, likely owing to more severe immunodeficiency (i.e., lower CD4^+^ cell counts and more commonly presenting with a history of opportunistic infections), and HBsAg loss [22^▪▪^]. Indeed, increased ALT levels at the start of HBV-active therapy and even after ART interruption have been associated with HBsAg loss in those with HIV/HBV, suggesting that liver inflammatory activity is likely more involved in clearing HBV than an immunoreconstitution effect *per se*[18^▪^,^23^,^24^^▪▪^].

In addition to HIV-related factors, other host factors have been inconsistently described as predictors of HBsAg loss. Studies have found both younger ages and older ages to be associated with HBsAg loss among PWH and PWoH with HBV [24^▪▪^,^25^–^27^]. This increased risk at the extremes of age has been confirmed in a recent meta-analysis including 102 studies with a total of more than 13 000 PWoH with HBV [28^▪^]. At the younger ages, immune response to HBV is less impaired and HBV infection duration much shorter [29,30]. Younger individuals are also more likely to have hepatic flares preceding HBsAg loss as a result of immune reconstitution-induced inflammatory syndrome [31]. At the older ages, HBV duration is much longer and perhaps higher rates of HBsAg loss are simply due to the natural course of infection. Female sex also appears as a beneficial factor for HBsAg loss early after initiating anti-HBV therapy in PWH and children without HIV [18^▪^,^19^,^27^], which suggests some pathophysiological mechanism with sex hormones or chromosomes. However, most other studies involving PWH or PWoH have not found such an association [12^▪^,^14^,20^▪^,^24^^▪▪^,32^▪^].

In terms of HBV virological factors, the probability of spontaneous and on-treatment HBsAg loss has been studied across different HBV genotypes in different populations of PWoH: individuals with HBV genotypes A and B appear to have a higher probability of HBsAg loss compared to those with C, D and E [33,34^▪^]. Although there are numerous studies examining the impact of HBV genotypes on treatment response in PWH [35,36^▪^], data relative to HBsAg loss are lacking in this population. In addition, mutations in the *precore* gene are associated with reduced rates of HBsAg loss in PWoH [37] and PWH [38]. Viral integration, which is in part linked to the intensity of viral replication and the duration of infection, may also decrease the likelihood of HBsAg loss by providing a persistent source of HBsAg expression [10^▪^].

TDF has been the mainstay of ART in PWH/HBV for the past two decades. TAF has been developed as an alternative tenofovir prodrug that can achieve higher concentrations of the active metabolite in cells with high activity of carboxyesterase and catepsin A, such as lymphocytes and hepatocytes [39]. A randomized controlled trial including ART-naïve PWH/HBV showed superior HBV DNA suppression with TAF-based ART compared to TDF-based ART after 48 weeks of therapy, but no significant differences after 96 weeks, also regarding HBsAg loss [40]. A recent cohort including, in part, participants from this trial showed indeed higher rates of HBsAg loss with TAF [24^▪▪^]. However, those data need to be confirmed in other studies involving different populations of PWH/HBV switching from TDF to TAF.

## THE POTENTIAL OF SERUM BIOMARKERS AS PREDICTORS OF HBsAg LOSS

Beyond serum markers assessed in clinical routine (i.e., ALT, HBV DNA, qualitative HBsAg and hepatitis B e antigen [HBeAg]), several novel biomarkers have raised interest as predictors of HBsAg loss. Among them, repeated quantitative HBsAg (qHBsAg) measurements have recently been included in the updated European Association for the Study of the Liver (EASL) guidelines as part of clinical routine, if available [8^▪▪^]. Other biomarkers including hepatitis B core-related antigen (HBcrAg) and HBV RNA, both of which appear to reflect the intrahepatic pool of transcriptionally active cccDNA, have raised interest and have been investigated in multiple, mainly retrospective, studies involving PWoH and PWH with HBV.

## QUANTITATIVE HBsAg

Most HBsAg found in serum is part of subviral particles, although HBsAg forms the lipid envelope surrounding the HBV nucleocapsid [41]. Low quantitative HBsAg levels at the start of anti-HBV therapy have been able to consistently predict HBsAg loss during therapy in both PWoH [26,42–44] and PWH [12,18,20^▪^,^24^^▪▪^,^45^–^49^]. Similarly, immediately declining qHBsAg levels after uptake of antiviral therapy can predict HBsAg loss in PWoH [26,50^▪^,51^▪^] and PWH [45,52,53^▪^]. It should be noted, however, that the individual trajectories of those who experience HBsAg loss are diverse and HBsAg loss may also occur after many years of antiviral therapy [50^▪^,53^▪^].

In HBeAg-negative PWH/HBV, qHBsAg levels appear to be associated with HBsAg loss only within 2 years, but not after longer periods [18^▪^]. This observation might be explained by the fact that most HBsAg is produced from iDNA, and not cccDNA, as treatment continues, while iDNA remains intact during therapy [54^▪^]. Current commercially available assays, such as the ARCHITECT HBsAg QT (Abbott Laboratories, Abbott Park, Illinois, USA) or the Elecsys HBsAg II Quant (Roche Diagnostics, Rotkreuz, Switzerland), cannot distinguish between the two sources of HBsAg. In recent years, assays attempting to quantify the different HBsAg isoforms (large, middle and small envelope proteins) have been developed and may help overcome this limitation [55^▪^,^56^]. However, this approach needs further validation and data involving PWH are lacking.

In addition, PWoH with residual low-level amounts of HBsAg (i.e., ≤0.05 IU/ml) who stop antiviral therapy are at an increased risk of HBsAg seroreversion [57^▪^]. Given that the sensitivity of the available quantitative assays is 0.05 IU/ml or less, the use of ultrasensitive qHBsAg assays with a detection limit of 0.005 IU/ml could provide a more accurate depiction of who could achieve sustained HBsAg loss, but a clinical use has yet to be determined [58,59^▪^].

## HEPATITIS B CORE-RELATED ANTIGEN

HBcrAg is a composite marker consisting of HBeAg, hepatitis B core antigen (HBcAg) and the p22cr protein, which are all products of the *precore* gene [60]. HBcrAg levels correlate with the transcriptionally active cccDNA reservoir in PWoH and PWH [61,62], which explains the interest in HBcrAg as a predictor of HBsAg loss during antiviral therapy. As expected purely from the composition of the marker, HBcrAg levels are higher in HBeAg-positive than in HBeAg-negative individuals not on antiviral therapy [63]. Accordingly, in a European multicohort collaboration, approximately 40% of HBeAg-negative PWH had undetectable HBcrAg levels before initiating ART with anti-HBV activity and during tenofovir-containing ART, most HBeAg-positive individuals remained HBcrAg-positive [18^▪^]. Nevertheless, HBcrAg levels usually decrease during tenofovir therapy in individuals with and without HBsAg loss [64^▪^,65^▪^], which already limits the discriminatory capacity for HBsAg loss. Interestingly, several studies showed detectable HBcrAg levels after HBsAg loss [18^▪^,64^▪^,^66^,^67^]. Whether this observation indicates persistently ongoing cccDNA transcription warrants further investigation.

There have been studies showing some predictive capacity of HBcrAg during therapy in individuals with HBV. A study involving children with HBV and without HIV identified HBcrAg decline during antiviral therapy as an independent predictor of HBsAg loss [68^▪^]. Low HBcrAg levels have also been found to be associated with spontaneous HBsAg loss in PWoH [69]. However, the clinical utility of HBcrAg levels at the start of anti-HBV therapy has been questioned by a recent systematic review including six studies with a total of 1257 PWoH [70]. In PWH, one recent study has shown a potential role to predict HBsAg loss within two years of tenofovir therapy in individuals who are HBeAg-negative [18^▪^]. However, the use of HBcrAg is limited not only by its nature as a composite biomarker, but also by the relatively high detection limit of 1000 U/ml of the only commercial assay available outside of Japan (Lumipulse G HBcrAg assay, Fujirebio Europe, Gent, Belgium): approximately one-third of HBeAg-negative PWH and PWoH with HBV have undetectable HBcrAg levels with the available assay [18^▪^,^71^]. Whether the novel iTACT-HBcrAg (Fujirebio, Tokyo, Japan) assay with higher sensitivity [72^▪^] or an assay detecting exclusively HBcAg [73] might overcome these limitations needs to be determined.

## CIRCULATING HEPATITIS B VIRUS RNA

HBV RNA circulating in the peripheral blood consists mainly of pregenomic RNA (pgRNA) [74], usually comprised in viral particles, as well as naked capsids and exosomes [75], but may also encompass spliced variants or truncated forms that may be expressed from integrated sequences [10^▪^]. Thus, the design of the assay for HBV RNA detection is critical for the correct interpretation of the results. As serum HBV pgRNA levels correlate closely with transcriptional activity of intrahepatic cccDNA [76^▪^,^77^], HBV pgRNA is an interesting biomarker reflecting residual cccDNA transcription. In recent years, several studies have investigated the potential of circulating HBV RNA as a predictor of HBsAg loss during antiviral therapy in PWoH [78–80] as well as PWH [18^▪^]. As many HBeAg-negative PWH already have undetectable HBV RNA levels at the start of tenofovir-containing ART, the predictive potential of HBV RNA could be limited to individuals who are HBeAg-positive [18^▪^].

Undetectable HBV RNA levels preceding HBsAg loss have been consistently described [64^▪^,^81^,^82^], but other studies have additionally found detectable HBV RNA levels at the time of HBsAg loss [83,84^▪^]. During tenofovir therapy, HBV RNA is known to decrease to undetectable levels in most PWH and PWoH, regardless of HBsAg loss [64^▪^]. However, initial declines in HBV RNA levels during tenofovir-containing ART have shown high sensitivity, but low specificity for HBsAg loss in PWH [64^▪^,65^▪^].

One important limitation of circulating HBV RNA as a biomarker is the lack of a standardized assay for its quantification, which prevents direct comparison between different studies and may explain discrepant results. Recent studies have mostly used either the Abbott Real Time HBV RNA Research Use Only assay (Abbott Diagnostics, Abbott Park, Illinois, USA) [85] or the COBAS HBV RNA assay (Roche Molecular Diagnostics, Pleasanton, California, USA) [86]. Other studies have also used in-house assays [84^▪^,^87^] that could be potentially measuring HBV RNA species from other sources than cccDNA as well. Recently, a molecular standard for HBV RNA detection and quantification has been proposed, but still requires further validation [88^▪^].

## IMMUNOLOGICAL MARKERS AS PREDICTORS

Hepatitis B core antibodies (HBcAb) are generally detectable in the serum of individuals who had contact with HBV and its quantification reflects intrahepatic immune activation, with the lowest levels found in resolved HBV infection [59^▪^,^89^]. Quantitative HBcAb (qHBcAb) levels decline during antiviral therapy [90,91] and lower qHBcAb levels have been found to be associated with HBsAg loss [92,93^▪^]. However, to our knowledge, only one study has evaluated qHBcAb trajectories in PWH [94].

A wide range of cytokines and chemokines are involved in the anti-HBV immune response. Among these, interferon-gamma-inducible protein 10 (IP-10) has been found to be the most promising as a predictor of HBsAg loss, particularly in PWoH during antiviral therapy [95,96,97^▪^]. However, to our knowledge, no study has investigated the predictive potential of IP-10 for HBsAg loss among PWH. Other soluble immune markers, including immunoinhibitory receptor programmed death-1 (PD-1), might be of interest, but have only been studied in small studies [21] or in the setting of trials investigating novel HBV agents [59^▪^].

Beyond serum markers, assessing the HBV-specific T- and B-cell responses, and differences in peripheral blood mononuclear cells (PBMCs) immune phenotypes might be able to predict HBsAg loss. In a cohort of 48 treatment-naive PWH/HBV, those with a >0.5 log_10_ IU/ml decline in qHBsAg levels within 6 months of ART start had higher T-cell activation and proliferation levels at baseline and after 1 year [98^▪▪^]. Distinct T cell immune profiles have been described in PWoH achieving HBsAg loss during pegylated interferon (peg-IFN) therapy [99,100,101^▪^]. Similarly, the presence of hepatitis B surface antibody (HBsAb)-specific B cells before starting peg-IFN treatment has been found to be associated with HBsAg loss [102^▪^]. However, it is not fully understood which aspects of the immune response are key to achieve HBsAg loss and clinical implementation in different settings might even be more complex than with the novel serum markers mentioned above.

## CONCLUSION

The interplay between host and virus is complex. Differences regarding the phase of HBV infection (HBeAg positive vs. negative) and treatment history make it difficult to pinpoint one single predictor of HBsAg loss. However, low qHBsAg levels and rapid initial declines in qHBsAg levels are consistently associated with HBsAg loss and regular assessments should be part of clinical routine. Other serum biomarkers, including HBcrAg and HBV RNA, could be useful in certain sub-populations and deliver insights into the mechanisms of novel HBV agents. Immunological markers might provide important data for the ongoing development of novel HBV treatment strategies.

## Acknowledgements

*None.*

### Financial support and sponsorship

*L.B. was supported by a Postdoc.Mobility fellowship from the Swiss National Science Foundation [P500PM_230336].*

### Conflicts of interest

*L.B. reports unrestricted research grants from Gilead Sciences and Roche Diagnostics, support for travel and conference participation from the CROI Foundation, and speaker honoraria from Roche, all paid to his institution and outside of the submitted work. A.B. reports receiving Speaker's fees from Gilead Sciences, Inc, outside of the submitted work.*

## REFERENCES AND RECOMMENDED READING

Papers of particular interest, published within the annual period of review, have been highlighted as:

▪ of special interest
▪▪ of outstanding interest
